# Supplementary material for: Glutathione-S-Transferase Theta 2 (GSTT2) Modulates the Response to Bacillus Calmette–Guérin Immunotherapy in Bladder Cancer Patients
Source: Int J Mol Sci. 2024 Aug 16;25(16):8947. doi: 10.3390/ijms25168947 (PMC11354831; doi:10.3390/ijms25168947)
Supplement: Supplementary file 1 [file ijms-25-08947-s001.zip › ijms-3135205-supplementary.pdf]

Supplementary Information

Glutathione-S-Transferase Theta 2 (GSTT2) Modulates the Response to Bacillus Calmette–Guérin Immunotherapy in Bladder Cancer Patients

Juwita N. Rahmat [1], Sin Mun Tham [1], Ting Li Ong [2], Yew Koon Lim [1], Mugdha Vijay Patwardhan [1], Lata Raman Nee Mani [1], Revathi Kamaraj [1], Yiong Huak Chan [2], Tsung Wen Chong [3,4], Edmund Chiong [1,5,*], Kesavan Esuvaranathan [1,5] and Ratha Mahendran [1,*]

[1] Department of Surgery, Yong Loo Lin School of Medicine, National University of Singapore, Singapore 119228, Singapore; juwita.r@nus.edu.sg (J.N.R.); surtsm@nus.edu.sg (S.M.T.); surlimy@nus.edu.sg (Y.K.L.); mugdha.p@u.nus.edu (M.V.P.); lraaman@gmail.com (L.R.N.M.); surpr@nus.edu.sg (R.K.); suresuva@nus.edu.sg (K.E.)
[2] School of Engineering, Biomedical Engineering, Temasek Polytechnic, Singapore 529757, Singapore; medcyh@nus.edu.sg (Y.H.C.)
[3] Department of Urology, Singapore General Hospital, Singapore 169608, Singapore; chong.tsung.wen@singhealth.com.sg
[4] Division of Surgery & Surgical Oncology, National Cancer Center Singapore, Singapore 168583, Singapore
[5] Department of Urology, National University Hospital, National University Health System, Singapore 119074, Singapore
* Correspondence: surce@nus.edu.sg (E.C.); surrm@nus.edu.sg (R.M.)

Supplementary Table S1. Primers annealing temperature and PCR product size.

Detected region	Primers 5′ to 3′ Forward and Reverse	Temperature °C	Product Size bp
GSTT2B deletion	CAC TCA ACA CAG TAG CCT CAT CGT G (GSTT2B-6858)<br>TGC CTC CCC TGC CTT ATT TC (GSTT2B-6857)<br>CCT TCT GAA ATG GAG CCT TTG (GSTT2B-2B)	55	847<br>and<br>505
-627	GCT CTG CAG GGG ACA GTC TG<br>TCT TAG AGT TTC ACC CAG TG	58	120
-537	CAC CTG CAA GTG TCA CAT CC<br>CAC GGA CAT CTG AGG GTT CGT	62	130
-277	ATC CCG AAA AGC AGA CCT<br>GTC GCT ATG AAC GCA CAG	55	145
17bp duplication	CTC TCT CAG TCT CGT CTA CTC C<br>CAC GGA CAT CTG AGG GTT CGT	54	590
GSTT1 deletion	CAGTTGTGAGCCACCGTACCC<br>CGATAGTTGCTGGCCCCCTC	72 *<br>(34 cycles)	1460
GSTT1 Full length	CCAGCTCACCGGATCATGGCCAG<br>R CCTTCCTTACTGGTCCTCACATCTC	63<br>(30 cycles)	466

* Combined annealing and elongation steps in one step (72℃; 45s).

A

B

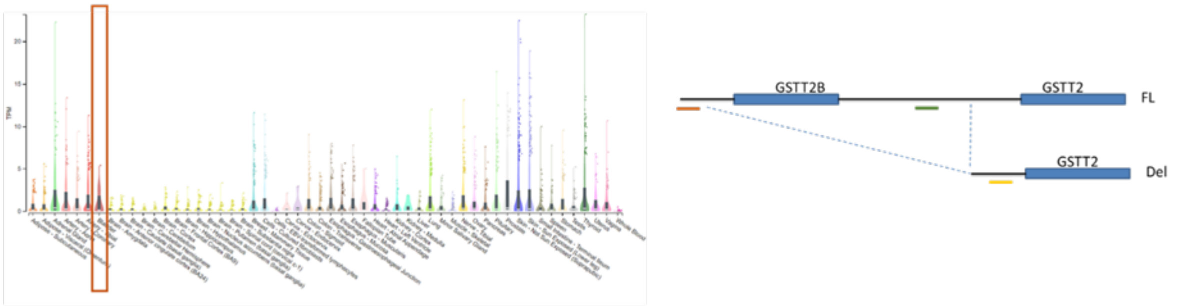

D

		GSTT2 promoter SNPs					
Cell Lines	GSTT2B	-627	-537	-277	-158	-127	GSTT1
MGH	FL	AA	GG	TT	GG	TT	Del
J82	FL	AA	GG	-	-	-	Del
T24	FL	AA	-	-	-	-	Del
RT4	FL/Del	AA	GA	-	-	-	Del
UMUC3	Del	GG	AA	TT	GG	TT	FL
U937	Del	GG	AA	TT	GG	TT	FL
THP1	FL/Del	GA	GA	TT	GG	TT	FL/Del
UMUC6	Del	GG	-	-	-	-	FL
UMUC9	Del	GG	-	-	-	-	FL
UMUC14	FL	AA	-	-	-	-	Del
HUC1	Fl/Del	GA	-	-	-	-	FL
HEK293T	FL	AA	-	-	-	-	Del

C

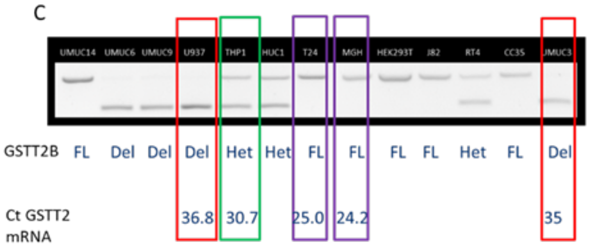

Supplementary Figure S1. GSTT status and expression in cancer cell lines and tissues. (**A**) GSTT2 expression in human tissues was downloaded from the GTex portal on the 24th of March 2022. (**B**) GSTT2B mapping strategy as described by Zhao Y et al., showing the location of the 3 primers. (**C**) Agarose gel of PCR products from GSTT2B deletion analysis using triple primers for PCR performed on DNA extracted from human bladder cancer cell lines (UMUC14, UMUC6, UMUC9, T24, MGH, J82, RT4, and UMUC3), promonocytic cell lines (U937 and THP1), a normal urothelial cell line (HUC1), human embryonic kidney cells (HEK293T) and genomic DNA from a peripheral blood sample (CC35). The GSTT2B genotype and real-time RNA analysis of GSTT2 expression post-stimulation of the cells with BCG are shown below the gel. Ct values greater than 32 are negative for gene expression. (**D**) Summary table showing the GSTT2B and GSTT1 genotypes (FL, FL/Del and Del) and GSTT2 promoter SNPs at positions -627, -537, -277, -158, -127 from the start site for the cell lines. A dash indicates that the genotype of the site was not determined.

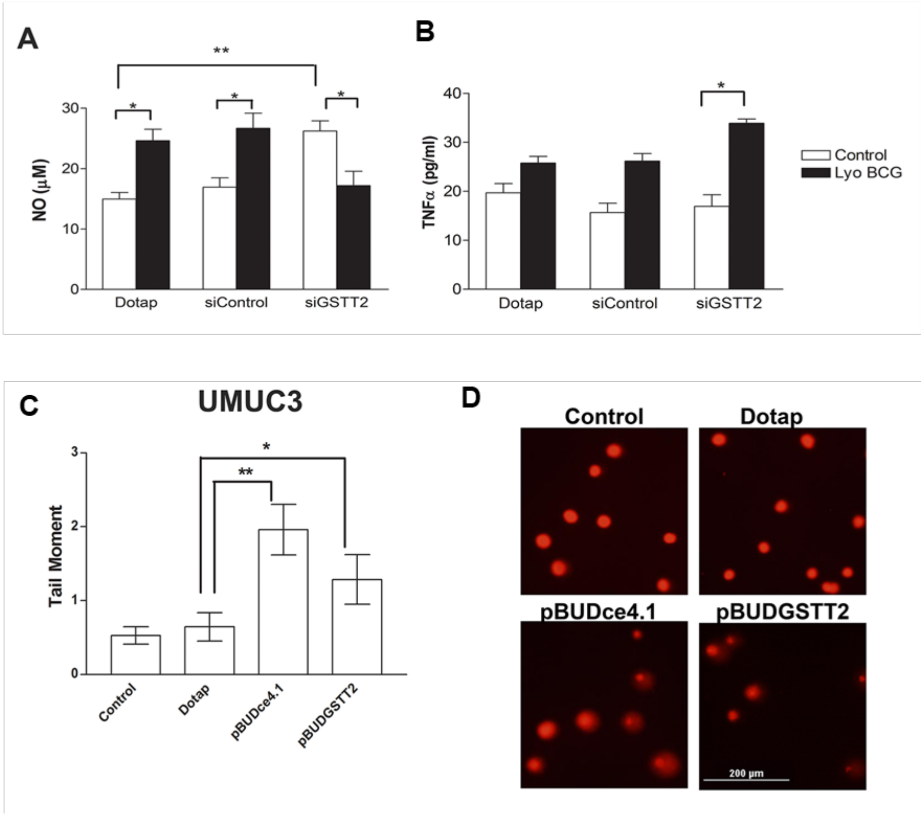

Supplementary Figure S2. GSTT2 modulation of cellular ROS and cytokine production after exposure to BCG. The effect of GSTT2 expression on (**A**) intracellular NO production and (**B**) TNFα levels in MGH cells exposed to lyophilized BCG. The effect of GSTT2 on DNA damage in UMUC3 cells. (**C**) Graph showing the measured tail moments and photographs of DNA comets observed in UMUC3 samples. (**D**) Fluorescence microscopy images of DNA stained from comet assay samples. Control non-transfected cells; cells exposed to DOTAP (transfection agent); cells transfected with pBudCe4.1 (empty vector) and cells transfected with pBudGSTT2. Experiments were performed twice in triplicates (n = 6). A minimum of 20 comets were analysed per sample. * represents a p-value <0.05 and ** a p-value <0.001.

Supplementary Table S2. Incidence of GSTT2 promoter SNPs and deletion.

Gene	Subjects	Genotypes			Total	p-value
GSTT2B		FL/FL	FL/Del	Del/Del		
	Control	37 (24.7%)	76 (50.7%)	37 (24.7%)	150	0.979
	Patients	50 (24.4%)	106(51.7%)	49(23.9%)	205	
Females	Controls	7 (20.0%)	24 (68.6%)	4 (11.4%)	35	0.069
	Patients	10 (28.9%)	16 (42.1%)	11 (29.7%)	37	
Males	Controls	30 (26.3%)	51 (44.7%)	33 (28.9%)	114	0.315
	Patients	38 (23.0%)	88 (53.3%)	39 (23.6%)	165	
-627		AA	GA	GG		
	Control	40 (26.7%)	77 (51.3%)	33 (22.0%)	150	0.835
	Patients	49 (23.9%)	110 (53.7%)	46 (22.4%)	205	
Females	Control	9 (25.2%)	23 (65.7%)	3 (8.6%)	35	0.165
	Patients	10 (27.0%)	18 (48.6%)	9 (24.3%)	37	
Males	Control	31 (27.2%)	53 (46.5%)	30 (26.3%)	114	0.394
	Patients	39 (23.2%)	92 (54.8%)	37 (22.0%)	168	
-537		AA	GA	GG		
	Control	36 (24.0%)	77 (51.3%)	37 (24.7%)	150	0.963
	Patients	50 (24.4%)	107 (52.2%)	45 (23.4%)	205	
Female	Control	3 (8.6%)	25 (71.4%)	7 (20.0%)	35	0.099
	Patients	9 (24.3%)	18 (48.6%)	10 (27.0%)	37	
-277		TT	TC	CC		
	Control	143 (95.3%)	7 (4.7%)	0 (0.0%)	150	0.547
	Patients	191 (93.2%)	13 (6.3%)	1 (0.5%)	205	
GSTT1		FL/FL	FL/Del	Del/Del		
	Control	23 (15.6%)	71 (48.3%)	53 (36.1%)	147	0.684
	Patients	26 (12.9%)	96(47.9%)	80 (39.6%)	202	
Female	Control	7 (21.2%)	10 (30.3%)	16 (48.5%)	33	0.084
	Patients	5 (13.5%)	21 (56.8%)	11 (29.7%)	37	
Male	Control	16 (14.2%)	61(54.0%)	36 (31.8%)	113	0.239
	Patients	21 (12.7%)	75 (45.5%)	69 (41.8%)	165	

Supplementary Table S3. Correlations of GSTT2B status and GSTT2 SNPs and GSTT1.

GSTT2B	-627			-537			-277			GSTT1		
	AA	GA	GG	AA	GA	GG	TT	TC	CC	FL/FL	Fl/Del	Del/Del
FL/FL	44 (88.0%)	6 (12.0%)	0 (0%)	0 (0%)	6 (12.0%)	44 (88.0%)	45 (90.0%)	4 (8.0%)	1 (2.0%)	3 (6.0%)	17 (34.0%)	30 (60.0%)
FL/Del	5 4.7%)	93 (87.7%)	8 (7.5%)	8 (7.5%)	94 (88.7%)	4 (3.8%)	99 (93.4%)	7 (6.6%)	0 (0%)	12 (11.7%)	50 (48.5%)	41 (39.8%)
Del/Del	0 (0%)	11 (22.4%)	38 (77.6%)	42 (85.7%)	7 (14.3%)	0 (0%)	47 (95.9%)	2 (4.1%)	0 (0%)	11 (22.4%)	29 (59.2%)	9 (18.4%)
p value	**<0.001**			**<0.001**			0.431			**0.001**		

Supplementary Table S4. GSTT2 SNPs and outcomes.

GSTT2	-627			-537			-277		
	AA	GA	GG	AA	GA	GG	TT	TC	CC
Recurrence									
No	28 (57.1%)	66 (60.0%)	29 (63.0%)	32 (64.0%)	65 (60.7%)	26 (54.2%)	114 (59.7%)	9 (60.2%)	0 (0%)
Yes	21 (42.9%)	44 (40.0%)	17 (37.0%)	18 (36.0%)	42 (39.3%)	22 (45.8%)	77 (40.3%)	4 (30.8%)	1 (100%)
p value	0.842			0.595			0.374		
Progression									
No	42 (85.7%)	100(90.0%)	39 (84.5%)	42 (84.0%)	98 (91.6%)	41 (85.4%)	168 (88.0%)	13 (100%)	0 (0%)
Yes	7 (14.3%)	10 (10.0%)	7 (11.7%)	8 (16.0%)	9 (8.4%)	7 (14.6%)	23 (12.0%)	0 (0%)	1 (100%)
p value	0.451			0.301			0.052		
Overall Survival									
No deaths	40 (81.6%)	87 (79.1%)	35 (76.1%)	38 (76.0%)	85 (79.4%)	39 (81.3%)	150 (78.5%)	11(84.6%)	
Yes	9 (18.4%)	23 (20.9%)	11 (23.9$)	12 (24/0%)	22 (20.6%)	9 (18.8%)	41 (21.5%)	2(15.4%)	
p value	0.802			0.806			0.603		

SNPs—Singel Nucleotide Polymorphisms, GSTT2—Glutathione-S-transferase theta 2.

Supplementary Table S5. Univariate and multivariate analysis of outcomes.

Univariate			
Factors	Recurrence<br>p-value HR (95% CI)	Progression<br>p-value HR (95% CI)	Survival
Age at diagnosis	0.058 1.022 (0.999-1.045)	0.019 1.05 (1.008-1.094)	**< 0.001** 1.092 (1.058-1.127)
Concomitant CIS	**0.03** 1.694 (1.053-2.723)	NS	0.066 1.688 (0.966-2.950)
Tumor grade 3	0.054 1.871 (0.990-3.535)	NS	NS
Previous history of multiple recurrences	**0.038** 2.048 (10.41-4.-29)	**0.026** 3.17 (1.149-8.745)	NS
Smoking	NS	NS	**0.006** 2.301 (1.264-4.187)
Multivariate Analysis*			
GSTT2B$^{FL/FL}$	**0.002** 5.523(1.827-16.693)	0.059 6.501 (0.932-45.374)	NS
GSTT2-537 AA	NS	**0.036** 35.419 (1.265-991.68)	NS
Age at diagnosis	NS	NS	**< 0.001** 1.099 (1.055-1.144)
Concomitant CIS	**0.003** 2.561 (2.389-4.725)	NS	NS
Tumor grade 3	NS	0.093 6.385 (0.735-55.461)	NS
Previous history of multiple recurrences	**0.008** 2.582 (1.278-5.218)	**0.009** 5.073 (1.487-17.305)	NS
Smoking	NS	NS	NS

* Age at diagnosis, race, sex, smoking history, GSTT2B status, promoter SNPs, stage, grade, number of BCG instillations, and previous history of recurrence NS—not significant, CIS—Carcinoma-in-situ, GSTT2—Glutathione-S-transferase theta 2.
